# Terminal decline in objective and self-reported measures of motor function before death: 10 year follow-up of Whitehall II cohort study

**DOI:** 10.1136/bmj.n1743

**Published:** 2021-08-05

**Authors:** Benjamin Landré, Aurore Fayosse, Céline Ben Hassen, Marcos D Machado-Fragua, Julien Dumurgier, Mika Kivimaki, Séverine Sabia, Archana Singh-Manoux

**Affiliations:** 1 Université de Paris, Inserm U1153, CRESS, Epidemiology of Ageing and Neurodegenerative diseases, Paris, France; 2 Cognitive Neurology Center, Lariboisière – Fernand Widal Hospital, AP-HP, Université de Paris, Paris, France; 3 Department of Epidemiology and Public Health, University College London, London, UK

## Abstract

**Objectives:**

To examine multiple objective and self-reported measures of motor function for their associations with mortality.

**Design:**

Prospective cohort study.

**Setting:**

UK based Whitehall II cohort study, which recruited participants aged 35-55 years in 1985-88; motor function component was added at the 2007-09 wave.

**Participants:**

6194 participants with motor function measures in 2007-09 (mean age 65.6, SD 5.9), 2012-13, and 2015-16.

**Main outcome measures:**

All cause mortality between 2007 and 2019 in relation to objective measures (walking speed, grip strength, and timed chair rises) and self-reported measures (physical component summary score of the SF-36 and limitations in basic and instrumental activities of daily living (ADL)) of motor function.

**Results:**

One sex specific standard deviation poorer motor function in 2007-09 (cases/total, 610/5645) was associated with an increased mortality risk of 22% (95% confidence interval 12% to 33%) for walking speed, 15% (6% to 25%) for grip strength, 14% (7% to 23%) for timed chair rises, and 17% (8% to 26%) for physical component summary score over a mean 10.6 year follow-up. Having basic/instrumental ADL limitations was associated with a 30% (7% to 58%) increased mortality risk. These associations were progressively stronger when measures were drawn from 2012-13 (mean follow-up 6.8 years) and 2015-16 (mean follow-up 3.7 years). Analysis of trajectories showed poorer motor function in decedents (n=484) than survivors (n=6194) up to 10 years before death for timed chair rises (standardised difference 0.35, 95% confidence interval 0.12 to 0.59; equivalent to a 1.2 (men) and 1.3 (women) second difference), nine years for walking speed (0.21, 0.05 to 0.36; 5.5 (men) and 5.3 (women) cm/s difference), six years for grip strength (0.10, 0.01 to 0.20; 0.9 (men) and 0.6 (women) kg difference), seven years for physical component summary score (0.15, 0.05 to 0.25; 1.2 (men) and 1.6 (women) score difference), and four years for basic/instrumental ADL limitations (prevalence difference 2%, 0% to 4%). These differences increased in the period leading to death for timed chair rises, physical component summary score, and ADL limitations.

**Conclusion:**

Motor function in early old age has a robust association with mortality, with evidence of terminal decline emerging early in measures of overall motor function (timed chair rises and physical component summary score) and late in basic/instrumental ADL limitations.

## Introduction

Ageing is characterised by a decline in cognitive and motor function over the adult life course,^1^ ^2^ ^3^ ^4^ along with an increase in heterogeneity of individual trajectories, partly as a result of pathological processes of age related chronic diseases.^5^ ^6^ In the years immediately preceding death, an accelerated decline in functioning has been observed,^7^ ^8^ referred to as “terminal decline.”^7^ As described in a recent review, terminal decline is observed in multiple domains, although much of the research is confined to cognitive decline.^9^

Better understanding of changes in functional status in the one or two years before death is useful for planning care, but it has minimal utility for identifying individuals who could benefit from clinical or behavioural interventions. Consideration of longer spans to study decline preceding death is also supported by findings showing decline in motor and cognitive function to be manifest starting in midlife.^2^ ^4^ Furthermore, several studies have shown midlife poorer cognitive and motor function to be associated with higher mortality risk.^10^ ^11^ ^12^ ^13^ The long term change in trajectories of functioning before death is less well characterised in relation to motor function. For cognitive function, long term trajectories are known, and change-point studies show that differences in different measures emerge up to 15 years before death.^14^

Change in motor function in the years before death is a dynamic process and may reflect changes over a longer period than at end of life examined in several studies.^15^ ^16^ ^17^ To date, few studies have considered a longer follow-up. An exception is a study showing decline in walking speed starting 10 years before death.^18^ Some studies have used composite measures of motor function,^16^ ^19^ ^20^ in which the role played by strength and upper and lower body function cannot be separated. A further limitation, apart from notable exceptions,^15^ is a lack of studies assessing both objective and self-reported measures of function. To overcome these limitations, the aim of this longitudinal cohort study was to examine multiple measures of motor function for their associations with mortality by using time-to-event analyses to capture the importance of between person differences in motor function and retrospective trajectory analyses to compare within person change in motor function over 10 years in survivors and deceased participants. Use of this twin analytical strategy allows both between person and within person differences in motor function to be examined in relation to mortality in the same study, with the second being reflected in the shape of the change in motor function leading to death.

## Methods

### Study population

The Whitehall II study is an ongoing prospective cohort of 10 308 British civil servants, 6895 men and 3413 women, aged 35-55 in 1985-88.^21^ Since baseline, follow-up clinical examinations have taken place approximately every four to five years using home based assessment for participants who choose this option and clinic based assessments (in London and major cities in the UK) for others; each wave takes approximately two years to complete. Measurement of motor function was introduced to the study at the 2007-09 clinical examination and repeated in 2012-13 and 2015-16 (flowchart in supplementary figure A). In addition to clinical examinations within the study, data over the follow-up are obtained via linkage to electronic health records of the UK National Health Service (NHS). The NHS provides most of the healthcare in the country, including inpatient and outpatient care, and record linkage is done using a unique NHS identifier held by all UK residents. Participants provided written informed consent at each wave.

### Motor function (2007-09, 2012-13, and 2015-16)

#### Objective measures

Walking speed was measured over an 8 ft (2.44 m) marked course, with no obstructions for an additional 2 ft at either end. Participants wore either low heeled, close fitting footwear or walked barefoot with instructions to “walk to the other end of the course at your usual walking pace, just as if you were walking down the street to go to the shops. Walk all the way past the other end of the tape before you stop.” Three tests were conducted, and a research nurse used a stopwatch to record the time taken to complete the test; we used the mean of three trials (m/s) in the analysis. Use of a walking stick, if habitual, was allowed.

Grip strength was measured using a Smedley hand grip dynamometer. Participants were seated, elbow on the table, forearm pointing upwards, and palm of the hand facing up. The dynamometer was adjusted to suit participants’ dominant hand and they were instructed to squeeze the dynamometer as hard as possible for two seconds. Three tests were performed with a one minute rest between tests; we used the maximum of these values in the analyses.^22^

Timed chair rises was the time (in seconds) to stand up and sit down five times. Participants started out sitting on an armless chair with feet resting on the floor and arms folded across the chest. They were instructed to stand up and sit down five times as quickly as possible without using their arms. To retain 275 participants with data on all other measures of motor function except timed chair rises, we imputed these data by using the sex specific mean score of the bottom fifth of performance as in a previous study.^23^

#### Self-reported measures

Self-reported functioning was measured using the physical component summary score of the Short Form 36 General Health Survey (SF-36).^24^ A low physical component summary score indicates limitations in self-care and daily activities, experiencing severe pain, and poor general health.

Self-reported functional limitations were assessed using difficulties in basic activities of daily living (ADL) and instrumental ADL.^25^ ^26^ Basic ADL were assessed by questions on the following six items: dressing, walking, bathing, eating, getting in bed, and using the toilet; instrumental ADLs included difficulty in cooking, shopping for groceries, making telephone calls, taking medication, doing housework, and managing money. Impaired functional status was determined by one or more limitations on a combined basic and instrumental ADL scale.

### Mortality

We defined death from any cause by using mortality records drawn from the British national mortality register (NHS Central Registry) until October 2019. The tracing exercise was carried out using the NHS identification number of each participant.

### Covariates

Sociodemographic variables included age, sex, ethnicity (white or non-white), marital status (living with a partner or single), and occupational position at age 50 (high, intermediate, or low, reflecting income and status at work).^21^ Health behaviours included smoking (never smoker, ex-smoker, current smoker), alcohol consumption (no alcohol in the previous week; moderate, 1-14 units/week; high, >14 units/week), time spent in moderate and vigorous physical activity (<150 minutes per week, at least the recommended amount of physical activity), and frequency of consumption of fruits and vegetables (less than daily, at least once a day). Body mass index, estimated using height and weight (kg/m^2^) assessed at the clinical examination, was categorised as <20, 20-24, 25-29.9, and ≥30.

We ascertained chronic diseases by using data from multiple sources, including clinical examinations in the study and linkage to electronic health records. We used three national databases: the national hospital episode statistics (HES) database with inpatient and outpatient data; the Mental Health Services Data Set, which in addition to inpatient and outpatient data also has data on care in the community; and the cancer registry. Chronic conditions considered were diabetes (fasting glucose ≥7.0 mmol/L, reported diabetes diagnosed by a doctor, use of diabetes medication, ICD-10 (international classification of diseases, 10th revision) codes E10-E14), coronary heart disease (12 lead resting electrocardiogram, ICD-10 codes I20-I25), stroke (MONICA-Ausburg stroke questionnaire, ICD-10 codes I60-I64), cancer (cancer registry with malignant cancer, ICD-10 codes C00-C97, to include colorectal, lung, breast, prostate, smoking related, and melanoma skin cancers), dementia (ICD-10 codes F00-F03, F05.1, G30, G31), Parkinson’s disease (self-report of longstanding illness, ICD-10 code G20), chronic obstructive pulmonary disease (self-report of longstanding illness, ICD-10 codes J41-J44), depression (self-report of longstanding illness, use of antidepressants, ICD-10 codes F32-F33), and arthritis (self-report of longstanding illness, ICD-10 codes M05, M06, M15-M19). We created a multimorbidity score as the count of these chronic conditions, ranging from 0 to 9.

### Statistical analysis

We standardised all continuous measures of motor function by using sex specific mean and standard deviation from baseline (2007-09). We examined the association between motor function and mortality in two ways: firstly using time-to-event analysis and then by comparison of retrospective trajectories of motor function over 10 years.

*Time-to-event analysis*—We used Cox proportional hazards regression to examine the association of motor function in 2007-09, 2012-13, and 2015-16 (separate models) with mortality. We used age as the time scale, left-truncated participants at age at assessment, and right-censored them at age of death or end of mortality follow-up (October 2019), whichever came first. We verified the proportional hazards assumption by plotting Schoenfeld residuals. Analyses were adjusted firstly for sociodemographic factors (sex, ethnicity, marital status, and occupational position at age 50) (model 1); additionally for health behaviours (physical activity, alcohol, tobacco, and fruit/vegetable consumption) (model 2), and then for body mass index and the multimorbidity score (model 3). Walking speed, grip strength, and SF-36 physical component summary score measures were rescaled so that hazard ratios associated with all continuous measure of motor function reflected the association with mortality for one standard deviation poorer performance. For basic/instrumental ADL limitations, the hazard ratio reflected having at least one limitation versus none.

*Retrospective analysis of motor function trajectories over 10 years*—We examined trajectories of motor function by using a backwards time scale such that time 0 was 31 December 2017 for survivors and date of death for participants who died between baseline (2007-09) and 31 December 2017. Deaths after this date were not considered in these analyses to restrict analyses on mortality occurring not long after the last measure of motor function. We defined retrospective trajectories by using linear mixed models for all motor function measures except basic/instrumental ADL limitations, for which we used logistic regression with generalised estimated equation and an unstructured correlation matrix. Time and time squared, and their interactions with age at time 0, sex, ethnicity, marital status, and occupation position were included in model 1; subsequent adjustment for covariates was the same as that in the fully adjusted Cox regression (model 3). Age was centred at the overall mean at time 0, and in the linear mixed models random effects for the intercept and time were used to allow for differences in motor function at the intercept (time 0) and change in motor function over time. We estimated the difference in motor function for the continuous measures and prevalence of basic/instrumental ADL limitations in survivors and decedents for each year, over the 10 years preceding end of follow-up or death, with a positive value indicating poorer motor function performance among decedents.

We used R software (version 4.0.3) for all analyses. Cox regression, linear mixed models, generalised estimated equation, and comparisons between survivors and decedents used the survival (version 3.2-7), nlme (version 3.1-149), geepack (version 1.3-2), and emmeans (version 1.5.2-1) packages, respectively. We reported estimates with 95% confidence intervals and considered two tailed P values to be significant at the 0.05 level.

### Additional analyses

In addition to considering the motor function measures separately in Cox regression in the main analyses, we did analyses including all motor function measures in the same model. Secondly, to examine the effect of missing data, we repeated the Cox regression analysis using inverse probability weighting to reflect the study population at recruitment (1985).^27^ This involved calculation of the probability of being included in the present study among participants who were alive by using data from baseline on sociodemographic factors and health behaviours as well as data on chronic conditions over the follow-up; then we used the inverse of these probabilities as weights in the Cox regression. Thirdly, we examined the role of chronic diseases in time-to-event analyses stratified by the status of multimorbidity at the assessment of motor function. Fourthly, we examined the possible influence of cognitive function by adding a measure of global cognition (the Mini Mental State Examination) as a covariate to the analyses. Fifthly, as basic and instrumental ADL were combined in the main analyses, we examined them separately to determine whether trends in long term terminal decline were similar in these two measures of functional limitations. Finally, in an alternative approach, we examined the association between change in motor function over the first two measures of motor function and subsequent mortality by using Cox regression and the same covariates as in the main analyses drawn from the 2012-13 assessments.

### Patient and public involvement

Participants of the Whitehall II study and members of the public were not involved in setting the research question or the outcome measures; nor were they involved in developing plans for recruitment, design, or implementation of the study. We recognise the importance of public involvement in instigating change in policy and practice, but funding for these activities was not available. The principal investigators of the Whitehall II study are in the process of seeking solutions to strengthen this aspect of the study. These analyses are part of a postdoctoral fellow’s research, for which no funds were available to consult or involve the public. Therefore, participants and members of the public could not be asked to contribute to interpretation or writing up of results before submission. We are grateful to the patient reviewer who made insightful suggestions that contributed to better contextualise our findings in the discussion section. All results are disseminated to study participants via newsletters and our website, which has a participant portal (https://www.ucl.ac.uk/epidemiology-health-care/research/epidemiology-and-public-health/research/whitehall-ii/participants-area ) and to a larger audience via media outreach.

## Results

Assessment of motor function was introduced to the study protocol at the 2007-09 wave of data collection when the age range of participants was 55-79 years and repeated in 2012-13 and 2015-16, leading to smaller numbers in analyses due to drop-out and mortality (supplementary figure A). The analyses of motor function trajectories were based on 6194 participants with data on at least one of three waves of motor function and the covariates. Compared with those excluded from these analyses (n=4114), participants included in the analyses were younger (44.0 *v* 45.6 years at recruitment in 1985-88) and were more likely to be men (72.0% (n=4459) *v* 59.2% (n=2436)), be white (92.5% (n=5729) *v* 83.9% (n=3452)), and have higher occupational position (43.2% (n=2674) *v* 25.1% (n=1034)).

Among the 6194 participants included in the analyses, 654 died between baseline (2007-09) and October 2019; the mean age at death was 76.8 (SD 6.2) years. Table 1 shows that participants who died were more likely to be older at baseline (mean age 69.7 *v* 65.1 years) and to have multimorbidity (27.2% (n=166) *v* 12.1% (n=610)) and poorer motor function compared with participants alive at the end of the follow-up. The motor function measures had a modest correlation with each other, ranging from 0.21 to 0.35 (see correlation matrix in supplementary table A).

**Table 1:** Population characteristics in 2007-09 by survival status at end of follow-up (October 2019). Values are numbers (percentages) unless stated otherwise

Characteristics	Total (n=5645)	Vital status at October 2019	
		Decedents (n=610)	Survivors (n=5035)
Mean (SD) age, years	65.6 (5.9)	69.7 (5.8)	65.1 (5.9)
Female sex	1539 (27.3)	152 (24.9)	1387 (27.5)
White	5244 (92.9)	570 (93.4)	4674 (92.8)
Married/cohabiting	4263 (75.5)	417 (68.4)	3846 (76.4)
High socioeconomic position	2476 (43.9)	239 (39.2)	2237 (44.4)
Moderate alcohol consumption	2901 (51.4)	277 (45.4)	2624 (52.1)
Never smoker	2722 (48.2)	249 (40.8)	2473 (49.1)
Daily fruit and vegetable consumption	2267 (40.2)	238 (39.0)	2029 (40.3)
Physical activity at recommended levels	3236 (57.3)	304 (49.8)	2932 (58.2)
Motor function:			
Mean (SD) walking speed, cm/s	110.6 (26.7)	101.1 (28.2)	111.8 (26.2)
Mean (SD) grip strength, kg	38.0 (10.6)	35.3 (10.5)	38.4 (10.6)
Mean (SD) timed chair rises, s	11.3 (3.4)	12.4 (4.2)	11.1 (3.3)
Mean (SD) SF-36 PCS score	48.8 (8.7)	45.3 (10.0)	49.2 (8.4)
Mean (SD) limitations in ADL or IADL	860 (15.2)	147 (24.1)	713 (14.2)
Mean (SD) body mass index	26.7 (4.4)	27.0 (4.8)	26.7 (4.4)
Chronic conditions:			
Diabetes	541 (9.6)	83 (13.6)	458 (9.1)
Coronary heart disease	1167 (20.7)	197 (32.3)	970 (19.3)
Stroke	216 (3.8)	60 (9.8)	156 (3.1)
Cancer	436 (7.7)	105 (17.2)	331 (6.6)
Dementia	7 (0.1)	3 (0.5)	4 (0.1)
Parkinson’s disease	20 (0.4)	7 (1.1)	13 (0.3)
Chronic obstructive pulmonary disease	47 (0.8)	16 (2.6)	31 (0.6)
Depression	561 (9.9)	69 (11.3)	492 (9.8)
Arthritis	496 (8.8)	68 (11.1)	428 (8.5)
Multimorbidity score*:			
0	3098 (54.9)	207 (33.9)	2891 (57.4)
1	1771 (31.4)	237 (38.9)	1534 (30.5)
≥2	776 (13.7)	166 (27.2)	610 (12.1)

ADL=activities of daily living; IADL=instrumental activities of daily living; SF-36 PCS score=Physical Component Summary score of Short Form 36 General Health Survey.

* Score is composed of chronic conditions listed above.

### Time-to-event analysis

We observed no sex differences in the association between measures of motor function and mortality; P values for the interaction term between sex and motor function measures ranged from 0.12 to 0.92. We therefore combined men and women in the analyses with sex specific standardisation of continuous motor function measures. Sex specific standard deviations correspond to a difference at baseline of 26.2 cm/s and 25.4 cm/s in walking speed, 8.5 kg and 6.2 kg in grip strength, 3.3 s and 3.6 s in timed chair rises, and 8.0 and 10.7 in physical component summary score among men and women respectively.

Poor performance on both objective and self-reported measures of motor function (1 SD lower performance in continuous measures and one or more limitations in basic/instrumental ADLs) was associated with higher risk of mortality (table 2) in analyses adjusted for sociodemographics (model 1) and health behaviours (model 2). This was the case for measures of motor function in 2007-09 (mean follow-up 10.6 (SD 1.8) years; mortality/total, 610/5645), in 2012-13 (mean follow-up 6.8 (1.0) years; mortality/total, 359/5083), and 2015-16 (mean follow-up 3.7 (0.6) years; mortality/total, 150/4440). Inclusion of body mass index and the multimorbidity score as covariates (model 3) attenuated associations, but poorer scores on all motor function measures remained associated with higher risk of mortality. The associations were stronger when follow-up was shorter; for example, one standard deviation slower walking speed was associated with a 22% (95% confidence interval 12% to 33%) higher risk of mortality when assessed in 2007-09 and a 49% (24% to 79%) higher risk when assessed in 2015-16.

**Table 2:** Association between standardised measures of motor function and subsequent mortality

	Hazard ratio (95% CI)		
	Model 1	Model 2	Model 3
**Motor function in 2007-09** * **†**			
Walking speed	1.32 (1.21 to 1.44)	1.28 (1.17 to 1.40)	1.22 (1.12 to 1.33)
Grip strength	1.22 (1.12 to 1.32)	1.19 (1.09 to 1.29)	1.15 (1.06 to 1.25)
Timed chair rises	1.21 (1.13 to 1.30)	1.19 (1.11 to 1.28)	1.14 (1.07 to 1.23)
SF-36 PCS score	1.30 (1.21 to 1.39)	1.26 (1.18 to 1.35)	1.17 (1.08 to 1.26)
Limitations in ADL or IADL	1.59 (1.31 to 1.91)	1.49 (1.23 to 1.80)	1.30 (1.07 to 1.58)
**Motor function in 2012-13** * **‡**			
Walking speed	1.50 (1.34 to 1.69)	1.45 (1.29 to 1.63)	1.38 (1.22 to 1.56)
Grip strength	1.21 (1.09 to 1.35)	1.19 (1.06 to 1.32)	1.14 (1.02 to 1.28)
Timed chair rises	1.28 (1.19 to 1.38)	1.26 (1.16 to 1.36)	1.20 (1.11 to 1.31)
SF-36 PCS score	1.31 (1.20 to 1.43)	1.26 (1.16 to 1.38)	1.17 (1.06 to 1.28)
Limitations in ADL or IADL	1.71 (1.36 to 2.14)	1.60 (1.27 to 2.01)	1.38 (1.09 to 1.74)
**Motor function in 2015-16** * §			
Walking speed	1.69 (1.42 to 2.01)	1.68 (1.40 to 2.00)	1.49 (1.24 to 1.79)
Grip strength	1.35 (1.14 to 1.61)	1.34 (1.13 to 1.59)	1.29 (1.08 to 1.53)
Timed chair rises	1.25 (1.15 to 1.35)	1.24 (1.14 to 1.34)	1.16 (1.06 to 1.27)
SF-36 PCS score	1.41 (1.24 to 1.59)	1.39 (1.23 to 1.58)	1.23 (1.07 to 1.40)
Limitations in ADL or IADL	2.13 (1.52 to 3.00)	2.08 (1.47 to 2.93)	1.58 (1.11 to 2.27)

ADL=activities of daily living; IADL=instrumental activities of daily living; SF-36 PCS score=Physical Component Summary score of Short Form 36 General Health Survey.

Model 1: adjusted for age, sex, ethnicity, marital status, and occupational position.

Model 2: model 1+health behaviours.

Model 3: model 2+body mass index categories and 9 point multimorbidity score.

* Hazard ratios for mortality associated with 1 SD sex specific poorer motor function, corresponding to 26.2 (25.4) cm/s slower walking speed, 8.5 (6.2) kg lower grip strength, 3.3 (3.6) more seconds to complete timed chair rises, and 8.0 (10.7) lower score in PCS in men (women). Limitations in ADL or IADL reflect having ≥1 limitations.

† Mortality/total, 610/5645; mean age, 65.6 (SD 5.9) years; mean follow-up, 10.6 (1.8) years.

‡ Mortality/total, 359/5083; mean age, 69.3 (5.7) years; mean follow-up, 6.8 (1.0) years.

§ Mortality/total, 150/4440; mean age, 72.1 (5.6) years; mean follow-up, 3.7 (0.6) years.

When all motor function measures were entered simultaneously in the Cox regression (supplementary table B), only slower walking speed was associated with higher mortality risk in the fully adjusted analyses (14% (4% to 25%) for the 2007-09 measure, 26% (10% to 44%) for the 2012-13 measure, and 29% (4% to 60%) for the 2015-16 measure of walking speed). Use of inverse probability weighting to account for missing data yielded results similar to those in the main analyses (supplementary table C). The association of motor function with mortality was similar in participants with and without multimorbidity (supplementary table D). Further adjustment for cognitive function did not alter the findings (supplementary table E).

Among the 4606 participants with motor function data in 2007-09 and 2012-13, assessments (supplementary table F; mortality, 316; mean age, 68.4 (5.7) years; mean follow-up, 7.0 (1.0) years), a one standard deviation decline in motor function over time was associated with an increased risk of mortality of 18% (5% to 32%) for walking speed, 22% (4% to 42%) for grip strength, and 16% (3% to 29%) for physical component summary score but not for timed chair rises (hazard ratio 1.08, 0.97 to 1.20). Compared with those with no basic/instrumental ADL limitations at these waves (mortality/total, 202/3567), participants who developed one limitation (mortality/total, 50/406) had a 37% (0% to 87%) higher risk of mortality.

### Retrospective trajectories of motor function over 10 years leading to death

A total of 484 deaths among 6194 participants were recorded between the start (2007-09 wave of data collection) and end of follow-up (31 December 2017). The end of follow-up in these analyses was earlier than in the Cox regression to restrict deaths contiguous to the last measure of motor function. Characteristics of these participants (supplementary table G) were similar to those in participants included in the time-to-event analysis.

**Fig 1:**
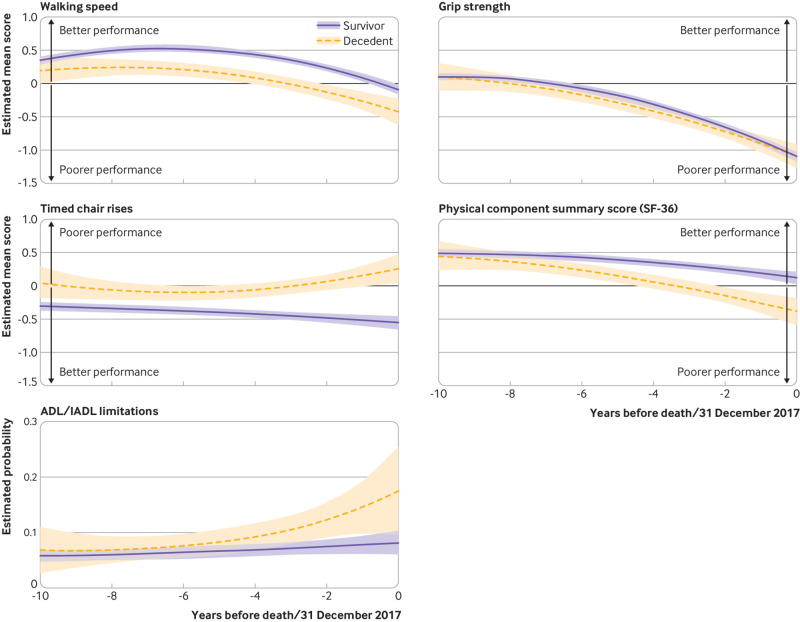
Trajectories of motor function over 10 years before death (decedents, n=484) and end of follow-up (survivors, n=5710). Estimated mean scores from linear mixed models (walking speed, grip strength, timed chair rises, physical component summary (PCS) score (SF-36)), and estimated probability from logistic regression (basic/instrumental activities of daily living (ADL/IADL) limitations) with generalised estimated equations. Analyses adjusted for age at year 0, sex, ethnicity, marital status, occupational position, vital status, time terms (time and time^2^), interactions of these covariates with time terms, and health behaviours, body mass index categories and 9 point multimorbidity score at motor function measurement. Higher scores on walking speed, grip strength, and SF-36 PCS score reflect better motor function; contrary is true for timed chair rises and ADL/IADL limitations. Sex specific standardised scores were used; 1 SD corresponded to 26.2 (25.4) cm/s for walking speed, 8.5 (6.2) kg for grip strength, 3.3 (3.6) more seconds for timed chair rises, and 8.0 (10.7) for PCS score in men (women)

Figure 1 shows the retrospective trajectories of motor function over the 10 years before death in decedents and before 31 December 2017 in those alive at this date; data are standardised mean scores for all measures except basic/instrumental ADLs, for which probabilities are presented in analyses adjusted for all covariates. The accompanying differences in each of the 10 years are shown adjusted for sociodemographic variables in supplementary table H and adjusted for all covariates in table 3.

**Table 3:** Differences in motor function between survivors and decedents in 10 years preceding death (mortality/total, 484/6194)* †

Years preceding death	Objective measures									Self-reported measures				
	Walking speed			Grip strength			Timed chair rises			SF-36 PCS score			ADL/IADL limitations	
	Difference in mean (95% CI)	P value		Difference in mean (95% CI)	P value		Difference in mean (95% CI)	P value		Difference in mean (95% CI)	P value		Difference in probabilities (95% CI)	P value
–10	0.16 (−0.06 to 0.38)	0.16		0.00 (−0.21 to 0.20)	0.98		0.35 (0.12 to 0.59)	0.003		0.03 (−0.19 to 0.25)	0.78		0.01 (−0.03 to 0.05)	0.61
–9	0.21 (0.05 to 0.36)	0.01		0.04 (−0.11 to 0.18)	0.62		0.30 (0.14 to 0.46)	<0.001		0.07 (−0.09 to 0.22)	0.39		0.01 (−0.02 to 0.03)	0.54
–8	0.25 (0.14 to 0.37)	<0.001		0.07 (−0.04 to 0.18)	0.23		0.27 (0.15 to 0.39)	<0.001		0.11 (−0.01 to 0.22)	0.05		0.01 (−0.01 to 0.03)	0.44
–7	0.29 (0.19 to 0.38)	<0.001		0.09 (−0.01 to 0.19)	0.07		0.26 (0.16 to 0.36)	<0.001		0.15 (0.05 to 0.25)	0.003		0.01 (−0.01 to 0.02)	0.32
–6	0.32 (0.22 to 0.41)	<0.001		0.10 (0.01 to 0.20)	0.04		0.27 (0.17 to 0.37)	<0.001		0.19 (0.09 to 0.29)	<0.001		0.01 (−0.01 to 0.03)	0.21
–5	0.34 (0.25 to 0.43)	<0.001		0.11 (0.01 to 0.21)	0.03		0.31 (0.20 to 0.41)	<0.001		0.24 (0.14 to 0.34)	<0.001		0.01 (0.00 to 0.03)	0.10
–4	0.35 (0.27 to 0.44)	<0.001		0.10 (0.01 to 0.20)	0.03		0.36 (0.26 to 0.46)	<0.001		0.29 (0.19 to 0.38)	<0.001		0.02 (0.00 to 0.04)	0.03
–3	0.36 (0.28 to 0.45)	<0.001		0.09 (0.00 to 0.18)	0.05		0.44 (0.34 to 0.54)	<0.001		0.34 (0.24 to 0.43)	<0.001		0.03 (0.01 to 0.05)	0.002
–2	0.36 (0.27 to 0.45)	<0.001		0.07 (−0.03 to 0.16)	0.17		0.54 (0.44 to 0.65)	<0.001		0.39 (0.29 to 0.49)	<0.001		0.04 (0.02 to 0.07)	<0.001
–1	0.36 (0.24 to 0.48)	<0.001		0.04 (−0.09 to 0.16)	0.57		0.67 (0.52 to 0.81)	<0.001		0.45 (0.31 to 0.58)	<0.001		0.06 (0.02 to 0.10)	0.001
0	0.35 (0.17 to 0.52)	<0.001		−0.01 (−0.18 to 0.17)	0.95		0.81 (0.61 to 1.02)	<0.001		0.51 (0.31 to 0.70)	<0.001		0.09 (0.02 to 0.16)	0.01
Difference in trajectories	0.20	-		0.50	-		<0.001	-		<0.001	-		0.04	-

ADL=activities of daily living; IADL=instrumental activities of daily living; SF-36 PCS score=Physical Component Summary score of Short Form 36 General Health Survey.

* Greater estimated mean difference reflects poorer motor function among decedents compared with survivors except for ADL/IADL limitations, which reflect probability of ≥1 limitations. One SD sex specific motor function corresponded to 26.2 (25.4) cm/s slower walking speed, 8.5 (6.2) kg lower grip strength, 3.3 (3.6) more seconds to complete timed chair rises, and 8.0 (10.7) lower score in PCS in men (women).

† Estimated from linear mixed models except ADL/IADL limitations, for which logistic regression with generalised estimated equation models were used; analyses adjusted for age at year 0, sex, ethnicity, marital status, occupational position, vital status, time terms (time and time^2^), interactions of sociodemographic covariates with time terms, and health behaviours, body mass index categories, and 9 point multimorbidity score.

In fully adjusted analyses (model 3, table 3), mean walking speed was faster in survivors than in decedents starting at nine years before death (difference in standardised walking speed 0.21 (95% confidence interval 0.05 to 0.36); equivalent to a difference of 5.5 cm/s and 5.3 cm/s in men and women respectively) and persisted to time 0. Grip strength in survivors was higher from year 6 (0.10 (0.01 to 0.20); equivalent to 0.9 (men) and 0.6 (women) kg difference) to year 3 (0.09 (0.00 to 0.18), 0.8 (men) and 0.6 (women) kg difference) before death. The shape of the overall 10 year trajectory of walking speed and grip strength (fig 1 and table 3) was similar in survivors and decedents.

Survivors had better performance than decedents on timed chair rises starting at year 10 (0.35 (0.12 to 0.59) SD; equivalent to a difference of 1.2 (men) and 1.3 (women) seconds), and this difference increased steadily with approach to time 0 (0.81 (0.61 to 1.02) SD; corresponding to 2.7 (men) and 2.9 (women) seconds). The physical component summary score was higher in survivors, indicating better motor function, starting from year 7 (0.15 (0.05 to 0.25) SD; equivalent to 1.2 (men) and 1.6 (women) score difference), and this difference increased over the period to time 0 (0.51 (0.31 to 0.70) SD; 4.1 (men) and 5.5 (women) score difference). The probability of basic/instrumental ADL limitation was lower in survivors starting from year 4 (0.02 (0.00 to 0.04)), with an increasing divergence to year 0 (0.09 (0.20 to 0.16)). Further examination of basic and instrumental limitations separately (supplementary table I) suggested that differences between survivors and decedents were due to ADL limitations. Adjustment for cognitive function did not alter the main findings (supplementary table J).

## Discussion

This study of repeated objective and self-reported measures of motor function spanning 10 years before death presents two key findings. Firstly, time-to-event analysis showed poorer motor function at mean age 65, 69, and 72 years to be associated with higher risk of mortality, with associations being stronger with later life measures of motor function. Secondly, trajectories of motor function over 10 years using a backwards time scale showed divergence, or terminal decline, in timed chair rises, physical component summary score (SF-36), and basic/instrumental ADL limitations starting 10, seven, and four years before death respectively. Given the definition of terminal decline as accelerated decline in functioning before death,^15^ or specifically divergence in trajectories of function, our results suggest important differences in terminal decline as a function of the specific measure of motor function. The difference between survivors and decedents in mean walking speed (from year 9 to year 0) and grip strength (from year 6 to year 3) did not change in the period leading to death. Differences in retrospective trajectories were greatest for timed chair rises and smallest for grip strength; increase in differences between survivors and decedents in the period leading to death was 4.7-fold in physical component summary score, 4.5-fold in basic/instrumental ADL limitations, and 2.3-fold in timed chair rises.

Use of the terminal decline framework allows better understanding of the relation between motor function and mortality owing to assessment of within person changes in motor function.^15^ ^28^ ^29^ Time-to-event analysis identifies the relevance of specific motor function measures, and the hazard ratio estimates reflect between person rather than within person differences in motor function. The originality of our approach is the use of retrospective trajectories, anchored to the date of death, so that distance to death is the same in those who died and survivors in comparisons of motor function. Increase in heterogeneity in individual trajectories is a hallmark of ageing^5^ ^6^; our analysis shows this heterogeneity to be meaningfully associated with mortality.

### Strengths and limitations of study

This study adds to the sparse literature on terminal decline in motor function and, to our knowledge, is the first to examine terminal and age related long term trajectories of multiple measures of motor function. The main strength of the study is the use of a twin approach, with modelling of trajectories along with Cox regression. The use of multiple measures of motor function, both objective and self-reported, is a further strength. The ability to consider a range of covariates in the analysis, including health behaviours, body mass index, and several chronic diseases, ensures that results are not driven by a certain behavioural or health profile.

The study findings need to be considered in light of some limitations. Firstly, we were not able to examine trajectories of motor function separately by cause of death owing to small number of deaths in categories of major causes of death. Some evidence suggests that the pattern of terminal decline differs according to cause of death.^30^ ^31^ Secondly, our findings are based on participants in early old age and may not be generalisable to deaths in the ninth and 10th decades of life. Thirdly, although a wide range of chronic conditions and health behaviours were included as covariates, acute events, such as falls or hospital admissions, also probably affect motor function trajectories. Fourthly, data are based on an occupational cohort at recruitment and participants were healthier than the general population, in terms of risk factors levels and incidence of disease. However, this does not necessarily affect risk factor-disease associations.^32^ For example, the associations of walking speed with mortality risk factors in Whitehall II, such as smoking, obesity, hypertension, and diabetes, are comparable to those found in 21 other cohort studies,^33^ ^34^ and the association between cardiovascular risk factors and incidence of cardiovascular disease in the Whitehall II study is similar to that in general population studies.^33^ Fifthly, the ethnicity distribution in the study reflects the UK population 30 years ago, and the study lacks sufficient numbers to allow analyses in specific minority groups.

### Comparison with previous studies

The overall results from time-to-event analyses in this study are consistent with the existing literature, despite differences in the manner in which motor function was considered in the analysis. A meta-analysis that compared the lowest and highest quarters of performance found grip strength (hazard ratio 1.67), walking speed (2.87), and chair rises (1.96) to be associated with higher risk of mortality.^35^ Most studies in the meta-analysis had a short follow-up and were based on participants older than 70 at baseline; the exception was grip strength, for which a wider range of data were available, and these studies show stronger associations with a shorter follow-up.^35^ Another pooled analysis of nine cohort studies (mean age of participants 73.5 years, mean follow-up 12.2 years) reported slower walking speed to be associated with increased mortality risk.^36^ In our study, repeat assessments of motor function show stronger associations when the follow-up was shorter, particularly for basic/instrumental ADL limitations.

The association of self-reported measures of motor function with mortality has mostly been examined using limitations in ADL in older adults, in whom it has a robust association with mortality,^37^ ^38^ ^39^ with follow-up ranging from one to >15 years. The evidence on physical functioning scales such as the physical component summary score from SF-36 is more limited; a recent meta-analysis of four studies with a mean follow-up of 1.8 years showed better scores to be associated with lower mortality risk (odds ratio for 1 unit increase: 0.95).^40^ In our study, both of these self-reported measures were associated with mortality, irrespective of the age at assessment. As with the objective measures of motor function, the hazard ratios of associations with mortality were higher when self-reported function was assessed closer to death.

Studies with repeat measures of motor function have shown decline in walking speed and grip strength in older adults to be associated with higher mortality risk in Cox regression.^41^ ^42^ ^43^ In this study, analysis of change in motor function between 2007-09 and 2012-13 found decline in both objective (walking speed, grip strength) and self-reported (physical component summary score and limitations in basic/instrumental ADLs) motor function to be associated with increased mortality risk (supplementary table F). However, this approach provides only a mean hazard ratio over the follow-up, which could vary from a few months to several years, rather than change in motor function in the years leading to death. A notable study on “fast” walking speed used a 10 year backwards time scale to show more rapid decline in decedents compared with survivors, but the authors did not do a formal comparison of differences in walking speed in the years leading to death.^18^ Previous studies have examined terminal decline in ADL limitations over the last few months or years before death.^31^ ^44^ ^45^ Our data show differences in basic/instrumental ADL limitations to be evident eight years and four years before death (supplementary table I) in analyses unadjusted for chronic conditions and fully adjusted respectively. Terminal decline in physical component summary score, a measure of overall physical functioning, bodily pain, and vitality,^24^ is rarely examined, and our results on divergence in trajectories four years before death in fully adjusted analyses suggest the usefulness of this measure to monitor motor function.

### Meaning of findings

Interest in objective measures of motor function is increasing, reflected in instruments such as the Short Physical Performance Battery (SPPB),^46^ composed of timed tests of standing balance, walking speed, and chair rises. Performance on this battery has a robust association with mortality.^19^ In this study, we chose to examine the association of objective and subjective measures of motor function, considering each measure separately, as use of a composite does not allow conclusions to be drawn on the importance of each component because results could be driven by one component or all measures might make a similar contribution. Furthermore, the SPPB does not include self-reported measures, which are easier to measure. Some authors have suggested that measures of upper body function, assessed using a handheld dynamometer, would add to the performance battery,^47^ but our data do not show substantial differences or terminal decline in grip strength. Our findings also highlight the importance of self-reported measures of motor function.

Motor function is controlled by central and peripheral structures in the nervous system, which include skeletal muscles and neural connections with muscle tissues. Decline in motor function preceding death is likely to be related to disease,^48^ anomalies in the physiological mechanisms of ageing,^49^ quantitative and qualitative changes in muscles,^50^ and more fundamental changes in mitochondria that contribute to accelerated ageing.^51^ Chronic diseases are thought to be important drivers of motor decline; in this study, adding the multimorbidity score to the analysis attenuated the associations in both time-to-event and backwards trajectories analyses. The importance of chronic diseases might be due to processes of chronic inflammation and oxidative stress; these are likely to operate across the lifecourse,^52^ as shown by diverging motor function trajectories before death in early old age in our study. However, in our analyses, the association between poorer motor function and increased mortality risk was also observed in participants free of multimorbidity.

### Conclusions

The ageing of populations worldwide makes understanding of the functional status of older adults and change in functioning with age important. Research on terminal decline is primarily on cognitive function,^9^ and when studies examine motor function the focus is on ADL limitations in the last few years of life. Our analysis of trajectories over 10 years in early old age shows the importance of objective and subjective measures of motor function. These results suggest that strategies to reduce accelerated decline should start before old age; early detection of changes in motor function might offer opportunities for prevention and targeted interventions.

## What is already known on this topic

Motor function declines with age, with considerable heterogeneity in the rate of decline
In older adults, functional limitations and poorer performance on measures of motor function are associated with higher risk of mortality
Limitations in activities of daily living become common in the last few months or years of life, but whether the difference in motor function trajectories spans a longer time frame is unknown

## What this study adds

Poor objective and self-reported motor function, assessed at mean age 65, 69, and 72 years, was associated with higher mortality risk; all associations were stronger with later life measures
Trajectories of motor function over 10 years using a backwards time scale showed terminal decline, increasingly poor motor function in decedents, starting 4-10 years before death
These findings suggest that early detection of changes in motor function might offer opportunities for prevention and targeted interventions

## Data Availability

Data sharing: Data, protocols, and other metadata of the Whitehall II study are available to the scientific community via either the Whitehall II study data sharing portal (https://www.ucl.ac.uk/epidemiology-health-care/research/epidemiology-and-public-health/research/whitehall-ii/data-sharing) or the DPUK platform (https://www.dementiasplatform.uk/).

## Web extra

Extra material supplied by authors

Web appendix: Supplementary materials

## Notes

Contributors: BL, SS, ASM, and AF developed the hypothesis and study design. BL and AF did the statistical analysis. BL wrote the first and successive drafts of the manuscript. All authors conceived and designed the study, analysed and interpreted the data, and drafted or critically revised the manuscript for important intellectual content, or, in addition, acquired data. ASM and MK obtained funding for the Whitehall II study. BL and AF had full access to the data and take responsibility for the integrity of the data and the accuracy of the data analysis. ASM and SS supervised the study and contributed equally to this work. BL is the guarantor. The corresponding author attests that all listed authors meet authorship criteria and that no others meeting the criteria have been omitted.

The lead author affirms that this manuscript is an honest, accurate, and transparent account of the study being reported; that no important aspects of the study have been omitted; and that any discrepancies from the study as planned (and, if relevant, registered) have been explained.

Dissemination to participants and related patient and public communities: The dissemination plan aims to target a wide audience, including members of the public, patients, and health professionals. It will be achieved using various channels: media outreach via press release from Inserm and University College London, scientific networks, and social media.

Provenance and peer review: Not commissioned; externally peer reviewed.

## References

[1] Whalley LJ Dick FD McNeill G . A life-course approach to the aetiology of late-onset dementias. Lancet Neurol 2006;5:87-96. 10.1016/S1474-4422(05)70286-6 16361026

[2] Singh-Manoux A Kivimaki M Glymour MM . Timing of onset of cognitive decline: results from Whitehall II prospective cohort study. BMJ 2012;344:d7622. 10.1136/bmj.d7622 22223828 PMC3281313

[3] Ferrucci L Cooper R Shardell M Simonsick EM Schrack JA Kuh D . Age-Related Change in Mobility: Perspectives From Life Course Epidemiology and Geroscience. J Gerontol A Biol Sci Med Sci 2016;71:1184-94. 10.1093/gerona/glw043 26975983 PMC4978365

[4] Dodds RM Syddall HE Cooper R . Grip strength across the life course: normative data from twelve British studies. PLoS One 2014;9:e113637. 10.1371/journal.pone.0113637 25474696 PMC4256164

[5] Brayne C . The elephant in the room - healthy brains in later life, epidemiology and public health. Nat Rev Neurosci 2007;8:233-9. 10.1038/nrn2091 17299455

[6] Kuh D Karunananthan S Bergman H Cooper R . A life-course approach to healthy ageing: maintaining physical capability. Proc Nutr Soc 2014;73:237-48. 10.1017/S0029665113003923 24456831 PMC3981474

[7] Wilson RS Yu L Leurgans SE Bennett DA Boyle PA . Proportion of cognitive loss attributable to terminal decline. Neurology 2020;94:e42-50. 10.1212/WNL.0000000000008671 31792096 PMC7011688

[8] Oliver D . David Oliver: “Progressive dwindling,” frailty, and realistic expectations. BMJ 2017;358:j3954. 10.1136/bmj.j3954 28874343

[9] Cohen-Mansfield J Skornick-Bouchbinder M Brill S . Trajectories of End of Life: A Systematic Review. J Gerontol B Psychol Sci Soc Sci 2018;73:564-72. 10.1093/geronb/gbx093 28977651

[10] Cooper R Strand BH Hardy R Patel KV Kuh D . Physical capability in mid-life and survival over 13 years of follow-up: British birth cohort study. BMJ 2014;348:g2219. 10.1136/bmj.g2219 24787359 PMC4004787

[11] Celis-Morales CA Welsh P Lyall DM . Associations of grip strength with cardiovascular, respiratory, and cancer outcomes and all cause mortality: prospective cohort study of half a million UK Biobank participants. BMJ 2018;361:k1651. 29739772 10.1136/bmj.k1651 PMC5939721

[12] Sabia S Guéguen A Marmot MG Shipley MJ Ankri J Singh-Manoux A . Does cognition predict mortality in midlife? Results from the Whitehall II cohort study. Neurobiol Aging 2010;31:688-95. 10.1016/j.neurobiolaging.2008.05.007 18541343 PMC2842015

[13] Davis D Cooper R Terrera GM Hardy R Richards M Kuh D . Verbal memory and search speed in early midlife are associated with mortality over 25 years’ follow-up, independently of health status and early life factors: a British birth cohort study. Int J Epidemiol 2016;45:1216-25. 10.1093/ije/dyw100 27498153 PMC6639118

[14] Karr JE Graham RB Hofer SM Muniz-Terrera G . When does cognitive decline begin? A systematic review of change point studies on accelerated decline in cognitive and neurological outcomes preceding mild cognitive impairment, dementia, and death. Psychol Aging 2018;33:195-218. 10.1037/pag0000236 29658744 PMC5906105

[15] Palmore E Cleveland W . Aging, terminal decline, and terminal drop. J Gerontol 1976;31:76-81. 10.1093/geronj/31.1.76 1081555

[16] Buchman AS Wilson RS Boyle PA Bienias JL Bennett DA . Change in motor function and risk of mortality in older persons. J Am Geriatr Soc 2007;55:11-9. 10.1111/j.1532-5415.2006.01032.x 17233680

[17] Diehr P Williamson J Burke GL Psaty BM . The aging and dying processes and the health of older adults. J Clin Epidemiol 2002;55:269-78. 10.1016/S0895-4356(01)00462-0 11864798

[18] Sabia S Dumurgier J Tavernier B Head J Tzourio C Elbaz A . Change in fast walking speed preceding death: results from a prospective longitudinal cohort study. J Gerontol A Biol Sci Med Sci 2014;69:354-62. 10.1093/gerona/glt114 23913931 PMC3976141

[19] Pavasini R Guralnik J Brown JC . Short Physical Performance Battery and all-cause mortality: systematic review and meta-analysis. BMC Med 2016;14:215. 10.1186/s12916-016-0763-7 28003033 PMC5178082

[20] Buchman AS Wilson RS Leurgans SE Bennett DA Barnes LL . Change in motor function and adverse health outcomes in older African-Americans. Exp Gerontol 2015;70:71-7. 10.1016/j.exger.2015.07.009 26209439 PMC4603389

[21] Marmot MG Smith GD Stansfeld S . Health inequalities among British civil servants: the Whitehall II study. Lancet 1991;337:1387-93. 10.1016/0140-6736(91)93068-K 1674771

[22] Haidar SG Kumar D Bassi RS Deshmukh SC . Average versus maximum grip strength: which is more consistent? J Hand Surg Br 2004;29:82-4. 10.1016/j.jhsb.2003.09.012 14734079

[23] Hurst L Stafford M Cooper R Hardy R Richards M Kuh D . Lifetime socioeconomic inequalities in physical and cognitive aging. Am J Public Health 2013;103:1641-8. 10.2105/AJPH.2013.301240 23865666 PMC3780680

[24] Ware JE Jr Kosinski M Bayliss MS McHorney CA Rogers WH Raczek A . Comparison of methods for the scoring and statistical analysis of SF-36 health profile and summary measures: summary of results from the Medical Outcomes Study. Med Care 1995;33(Suppl):AS264-79. 7723455

[25] Katz S Downs TD Cash HR Grotz RC . Progress in development of the index of ADL. Gerontologist 1970;10:20-30. 10.1093/geront/10.1_Part_1.20 5420677

[26] Lawton MP Brody EM . Assessment of older people: self-maintaining and instrumental activities of daily living. Gerontologist 1969;9:179-86. 10.1093/geront/9.3_Part_1.179 5349366

[27] Rusmaully J Dugravot A Moatti JP . Contribution of cognitive performance and cognitive decline to associations between socioeconomic factors and dementia: A cohort study. PLoS Med 2017;14:e1002334. 10.1371/journal.pmed.1002334 28650972 PMC5484463

[28] Piccinin AM Muniz G Matthews FE Johansson B . Terminal decline from within- and between-person perspectives, accounting for incident dementia. J Gerontol B Psychol Sci Soc Sci 2011;66:391-401. 10.1093/geronb/gbr010 21389088 PMC3132263

[29] MacDonald SW Hultsch DF Dixon RA . Aging and the shape of cognitive change before death: terminal decline or terminal drop? J Gerontol B Psychol Sci Soc Sci 2011;66:292-301. 10.1093/geronb/gbr001 21300703 PMC3078759

[30] Lunney JR Albert SM Boudreau R Ives D Newman AB Harris T . Fluctuating Physical Function and Health: Their Role at the End of Life. J Palliat Med 2019;22:424-6. 10.1089/jpm.2018.0289 30570377 PMC6459261

[31] Lunney JR Lynn J Foley DJ Lipson S Guralnik JM . Patterns of functional decline at the end of life. JAMA 2003;289:2387-92. 10.1001/jama.289.18.2387 12746362

[32] Rothman KJ Gallacher JE Hatch EE . Why representativeness should be avoided. Int J Epidemiol 2013;42:1012-4. 10.1093/ije/dys223 24062287 PMC3888189

[33] Batty GD Shipley M Tabák A . Generalizability of occupational cohort study findings. Epidemiology 2014;25:932-3. 10.1097/EDE.0000000000000184 25265141

[34] Stringhini S Carmeli C Jokela M LIFEPATH Consortium . Socioeconomic status, non-communicable disease risk factors, and walking speed in older adults: multi-cohort population based study. BMJ 2018;360:k1046. 10.1136/bmj.k1046 29572376 PMC5865179

[35] Cooper R Kuh D Hardy R Mortality Review Group FALCon and HALCyon Study Teams . Objectively measured physical capability levels and mortality: systematic review and meta-analysis. BMJ 2010;341:c4467. 10.1136/bmj.c4467 20829298 PMC2938886

[36] Studenski S Perera S Patel K . Gait speed and survival in older adults. JAMA 2011;305:50-8. 10.1001/jama.2010.1923 21205966 PMC3080184

[37] Gobbens RJJ van der Ploeg T . The Prediction of Mortality by Disability Among Dutch Community-Dwelling Older People. Clin Interv Aging 2020;15:1897-906. 10.2147/CIA.S271800 33116444 PMC7547136

[38] Walter LC Brand RJ Counsell SR . Development and validation of a prognostic index for 1-year mortality in older adults after hospitalization. JAMA 2001;285:2987-94. 10.1001/jama.285.23.2987 11410097

[39] Nascimento CM Oliveira C Firmo JOA Lima-Costa MF Peixoto SV . Prognostic value of disability on mortality: 15-year follow-up of the Bambuí cohort study of aging. Arch Gerontol Geriatr 2018;74:112-7. 10.1016/j.archger.2017.10.011 29096224

[40] Phyo AZZ Freak-Poli R Craig H . Quality of life and mortality in the general population: a systematic review and meta-analysis. BMC Public Health 2020;20:1596. 10.1186/s12889-020-09639-9 33153441 PMC7646076

[41] Andrasfay T . Changes in Physical Functioning as Short-Term Predictors of Mortality. J Gerontol B Psychol Sci Soc Sci 2020;75:630-9. 10.1093/geronb/gby133 30388248 PMC7768692

[42] Granic A Davies K Jagger C M Dodds R Kirkwood TBL Sayer AA . Initial level and rate of change in grip strength predict all-cause mortality in very old adults. Age Ageing 2017;46:970-6. 10.1093/ageing/afx087 28541466 PMC5860048

[43] Syddall HE Westbury LD Dodds R Dennison E Cooper C Sayer AA . Mortality in the Hertfordshire Ageing Study: association with level and loss of hand grip strength in later life. Age Ageing 2017;46:407-12. 10.1093/ageing/afw222 27932364 PMC5500162

[44] Lunney JR Albert SM Boudreau R Health ABC study . Three Year Functional Trajectories Among Old Age Survivors and Decedents: Dying Eliminates a Racial Disparity. J Gen Intern Med 2018;33:177-81. 10.1007/s11606-017-4232-6 29204976 PMC5789114

[45] Lunney JR Albert SM Boudreau R Health Aging and Body Composition Study . Mobility Trajectories at the End of Life: Comparing Clinical Condition and Latent Class Approaches. J Am Geriatr Soc 2018;66:503-8. 10.1111/jgs.15224 29345750 PMC5849481

[46] Guralnik JM Simonsick EM Ferrucci L . A short physical performance battery assessing lower extremity function: association with self-reported disability and prediction of mortality and nursing home admission. J Gerontol 1994;49:M85-94. 10.1093/geronj/49.2.M85 8126356

[47] Mijnarends DM Meijers JM Halfens RJ . Validity and reliability of tools to measure muscle mass, strength, and physical performance in community-dwelling older people: a systematic review. J Am Med Dir Assoc 2013;14:170-8. 10.1016/j.jamda.2012.10.009 23276432

[48] Kalyani RR Corriere M Ferrucci L . Age-related and disease-related muscle loss: the effect of diabetes, obesity, and other diseases. Lancet Diabetes Endocrinol 2014;2:819-29. 10.1016/S2213-8587(14)70034-8 24731660 PMC4156923

[49] López-Otín C Blasco MA Partridge L Serrano M Kroemer G . The hallmarks of aging. Cell 2013;153:1194-217. 10.1016/j.cell.2013.05.039 23746838 PMC3836174

[50] Goodpaster BH Park SW Harris TB . The loss of skeletal muscle strength, mass, and quality in older adults: the health, aging and body composition study. J Gerontol A Biol Sci Med Sci 2006;61:1059-64. 10.1093/gerona/61.10.1059 17077199

[51] Sun N Youle RJ Finkel T . The Mitochondrial Basis of Aging. Mol Cell 2016;61:654-66. 10.1016/j.molcel.2016.01.028 26942670 PMC4779179

[52] Blodgett JM Cooper R Davis DHJ Kuh D Hardy R . Associations Between Factors Across Life and One-Legged Balance Performance in Mid and Later Life: Evidence From a British Birth Cohort Study. Front Sports Act Living 2020;2020:00028. 10.3389/fspor.2020.00028 32395714 PMC7212024

