# Supplementary material for: Terminal decline in objective and self-reported measures of motor function before death: 10 year follow-up of Whitehall II cohort study
Source: BMJ. 2021 Aug 5;374:n1743. doi: 10.1136/bmj.n1743 (PMC8336001; doi:10.1136/bmj.n1743)
Supplement: Supplementary file 1 — Web appendix: Supplementary materials [file lanb065492.ww1.pdf]

Terminal decline in objective and self-reported measures of motor function before death: 10-year follow-up of Whitehall II cohort study.

Benjamin Landré, postdoctoral fellow[1]¤,

Aurore Fayosse, statistician[1],

Céline Ben Hassen, postdoctoral fellow[1],

Marcos D. Machado-Fragua, postdoctoral fellow[1],

Julien Dumurgier, associate professor[1,2],

Mika Kivimaki, professor[3],

Séverine Sabia, research associate[1,3],

Archana Singh-Manoux, professor[1,3]

[1]Université de Paris, Inserm U1153, CRESS, Epidemiology of Ageing and Neurodegenerative diseases, Paris, France

[2]Cognitive Neurology Center, Lariboisière – Fernand Widal Hospital, AP-HP, Université de Paris, Paris, France

[2]Department of Epidemiology and Public Health, University College London, UK

SUPPLEMENTAL DATA

Supplementary figure A. Flow chart of the study.

Supplementary table A. Correlation matrix of measures of motor function.

Supplementary table B. Association between motor function and mortality in mutually adjusted models.

Supplementary table C. Association between standardized measures of motor function and subsequent mortality, using inverse probability weighting to account for missing data.

Supplementary table D. Association between standardized measures of motor function and subsequent mortality as a function of multimorbidity status at assessment of motor function.

Supplementary table E. Association between standardized measures of motor function and subsequent mortality, with addition adjustment for global cognition (Mini Mental State Examination).

Supplementary table F. Association of decline in motor function performance between 2007-2009 and 2012-2013* and subsequent mortality.

Supplementary table G. Population characteristics in 2007-2009 by survival status at time 0 in the retrospective time scale in the analysis (date of death or 31st of December 2017).

Supplementary table H. Differences in motor function between survivors and decedents in the 10 years preceding death in analysis adjusted only for sociodemographic variables, N mortality/N total = 484/6,194. * †

Supplementary table I. Difference in the probability of limitations in ADL and IADL, examined separately, between survivors and decedents in the 10 years preceding death, N mortality/N total = 484/6,194.*

Supplementary table J. Difference in motor function between survivors and decedents in the 10 years preceding death with additional adjustment for global cognition (Mini Mental State Examination), N mortality/N total = 477/6,149.* †

Supplementary figure A. Flow-chart of the study.

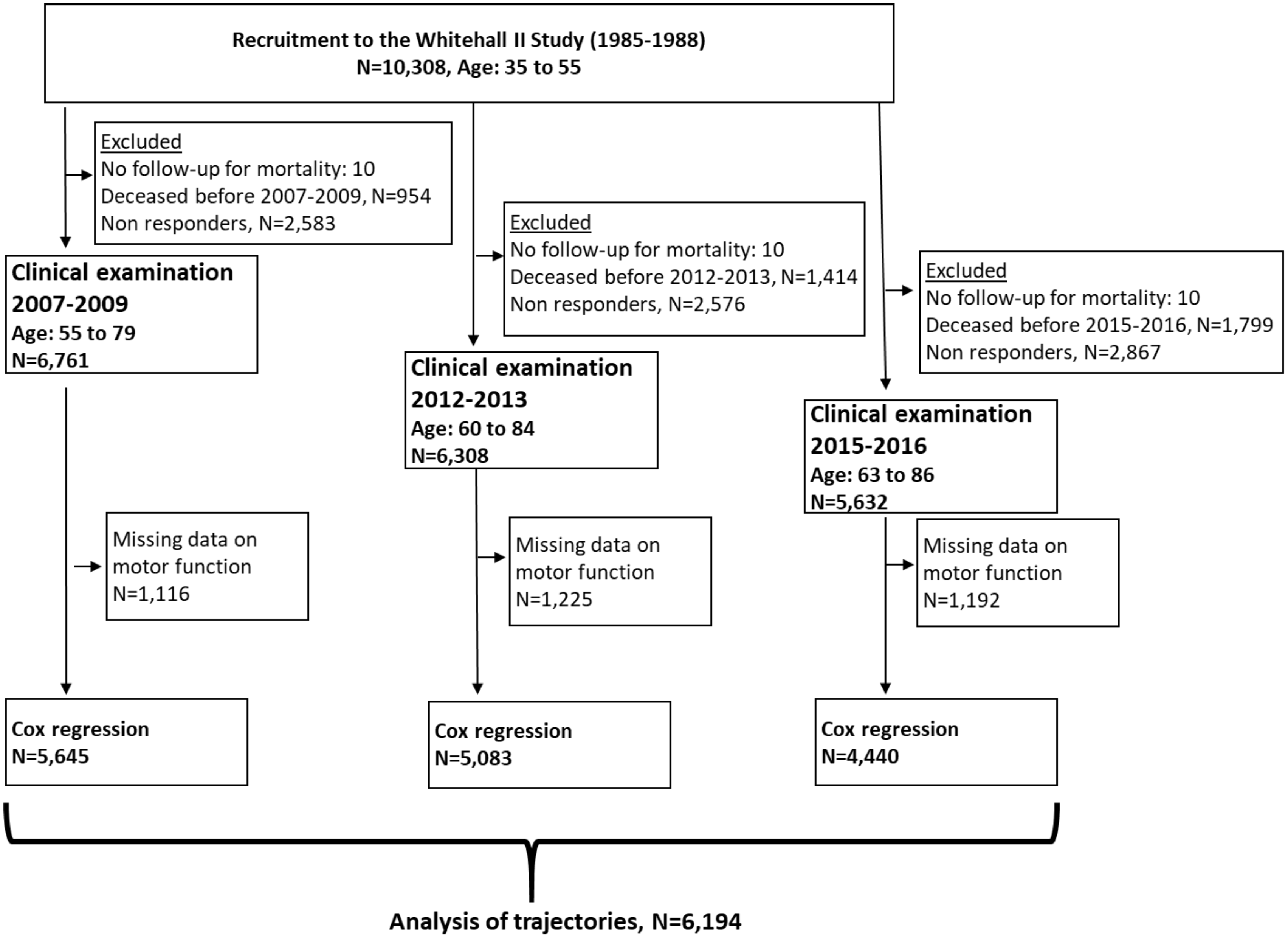

Supplementary table A. Correlation matrix of measures of motor function.

	Walking speed	Grip strength	Chair rise time	SF-36 PCS score
**Walking speed**	x			
**Grip strength**	0.29	x		
**Chair rise time**	-0.34	-0.22	x	
**SF-36 PCS score**	0.35	0.21	-0.35	x

Abbreviation: SF-36 PCS score: Physical Component Summary score of the Short Form 36 General Health Survey.

Supplementary table B. Association between motor function and mortality in mutually adjusted models.

	**Model 1**	**Model 2**	**Model 3**
	HR (95% CI)	HR (95% CI)	HR (95% CI)
**Motor function in 2007-2009*** †			
Walking speed	1.16 (1.06 to 1.28)	1.15 (1.05 to 1.26)	1.14 (1.04 to 1.25)
Grip strength	1.12 (1.02 to 1.22)	1.11 (1.02 to 1.21)	1.09 (1.00 to 1.19)
Timed chair rises	1.09 (1.01 to 1.17)	1.08 (1.00 to 1.17)	1.07 (0.99 to 1.16)
SF-36 PCS score	1.18 (1.08 to 1.28)	1.16 (1.06 to 1.26)	1.09 (1.00 to 1.19)
Limitations in ADL or IADL	1.09 (0.87 to 1.37)	1.08 (0.87 to 1.36)	1.07 (0.85 to 1.34)
**Motor function in 2012-2013*** ‡			
Walking speed	1.27 (1.12 to 1.45)	1.25 (1.10 to 1.43)	1.26 (1.10 to 1.44)
Grip strength	1.09 (0.97 to 1.21)	1.09 (0.97 to 1.21)	1.06 (0.95 to 1.19)
Timed chair rises	1.14 (1.03 to 1.25)	1.14 (1.03 to 1.25)	1.12 (1.02 to 1.23)
SF-36 PCS score	1.13 (1.01 to 1.26)	1.10 (0.99 to 1.23)	1.05 (0.94 to 1.17)
Limitations in ADL or IADL	1.11 (0.84 to 1.46)	1.10 (0.83 to 1.45)	1.06 (0.80 to 1.40)
**Motor function in 2015-2016*** §			
Walking speed	1.32 (1.07 to 1.62)	1.32 (1.07 to 1.63)	1.29 (1.04 to 1.60)
Grip strength	1.20 (1.00 to 1.43)	1.20 (1.01 to 1.43)	1.18 (0.99 to 1.41)
Timed chair rises	1.10 (0.99 to 1.22)	1.10 (0.99 to 1.22)	1.07 (0.96 to 1.19)
SF-36 PCS score	1.14 (0.97 to 1.35)	1.14 (0.96 to 1.34)	1.05 (0.88 to 1.24)
Limitations in ADL or IADL	1.28 (0.83 to 1.96)	1.29 (0.84 to 1.97)	1.20 (0.78 to 1.83)

Abbreviations: ADL: Activities of Daily Living; IADL: Instrumental Activities of Daily Living; SF-36 PCS score: Physical Component Summary score of the Short Form 36 General Health Survey; HR: Hazard ratio; CI: Confidence interval.

* HRs for mortality associated with 1 SD sex-specific poorer motor function, corresponding to 26.2 (25.4) cm/s slower walking speed, 8.5 (6.2) kg lower grip strength, 3.3 (3.6) more seconds to undertake timed chair rises and 8.0 (10.7) lower score in PCS in men (women) respectively. Limitations in ADL or IADL reflects having 1 or more limitations.

†N mortality/N total = 610/5,645; Mean (SD) age, 65.6 (5.9) years; Mean (SD) follow-up, 10.6 (1.8) years.

‡N mortality/N total = 359/5,083; Mean (SD) age, 69.3 (5.7) years; Mean (SD) follow-up, 6.8 (1.0) years.

§N mortality/N total = 150/4,440; Mean (SD) age, 72.1 (5.6) years; Mean (SD) follow-up, 3.7 (0.6) years.

Model 1: adjusted for age, sex, ethnicity, marital status and occupational position.

Model 2: Model 1 + health behaviours.

Model 3: Model 2 + BMI categories and 9-point multimorbidity score.

Supplementary table C. Association between standardized measures of motor function and subsequent mortality, using inverse probability weighting to account for missing data.

	**Model 1**<br>HR (95% CI)	**Model 2**<br>HR (95% CI)	**Model 3**<br>HR (95% CI)
**Motor function in 2007-2009*** †			
Walking speed	1.37 (1.25 to 1.51)	1.33 (1.21 to 1.46)	1.27 (1.15 to 1.39)
Grip strength	1.24 (1.13 to 1.36)	1.21 (1.10 to 1.32)	1.17 (1.07 to 1.28)
Timed chair rises	1.20 (1.12 to 1.29)	1.18 (1.10 to 1.27)	1.13 (1.05 to 1.22)
SF-36 PCS score	1.29 (1.20 to 1.39)	1.25 (1.17 to 1.35)	1.17 (1.08 to 1.27)
Limitations in ADL or IADL	1.63 (1.34 to 1.98)	1.54 (1.27 to 1.87)	1.36 (1.11 to 1.66)
**Motor function in 2012-2013*** ‡			
Walking speed	1.53 (1.34 to 1.74)	1.47 (1.28 to 1.68)	1.40 (1.22 to 1.60)
Grip strength	1.20 (1.07 to 1.35)	1.18 (1.05 to 1.32)	1.13 (1.01 to 1.28)
Timed chair rises	1.27 (1.18 to 1.36)	1.25 (1.16 to 1.34)	1.20 (1.12 to 1.30)
SF-36 PCS score	1.29 (1.18 to 1.42)	1.25 (1.14 to 1.37)	1.16 (1.04 to 1.28)
Limitations in ADL or IADL	1.73 (1.36 to 2.19)	1.61 (1.27 to 2.04)	1.39 (1.10 to 1.76)
**Motor function in 2015-2016*** §			
Walking speed	1.79 (1.49 to 2.16)	1.79 (1.48 to 2.16)	1.58 (1.31 to 1.92)
Grip strength	1.38 (1.13 to 1.67)	1.37 (1.13 to 1.65)	1.31 (1.09 to 1.58)
Timed chair rises	1.24 (1.15 to 1.34)	1.24 (1.14 to 1.34)	1.17 (1.06 to 1.28)
SF-36 PCS score	1.40 (1.23 to 1.59)	1.39 (1.22 to 1.58)	1.22 (1.06 to 1.40)
Limitations in ADL or IADL	2.01 (1.41 to 2.87)	1.97 (1.38 to 2.82)	1.47 (1.02 to 2.13)

Abbreviations: ADL: Activities of Daily Living; IADL: Instrumental Activities of Daily Living; SF-36 PCS score: Physical Component Summary score of the Short Form 36 General Health Survey; HR: Hazard ratio; CI: Confidence interval.

*HRs for mortality associated with 1 SD sex-specific poorer motor function, corresponding to 26.2 (25.4) cm/s slower walking speed, 8.5 (6.2) kg lower grip strength, 3.3 (3.6) more seconds to undertake timed chair rises and 8.0 (10.7) lower score in PCS in men (women) respectively. Limitations in ADL or IADL reflects having 1 or more limitations.

†N mortality/N total = 610/5,645; Mean (SD) age, 65.6 (5.9) years; Mean (SD) follow-up, 10.6 (1.8) years.

‡N mortality/N total = 359/5,083; Mean (SD) age, 69.3 (5.7) years; Mean (SD) follow-up, 6.8 (1.0) years.

§N mortality/N total = 150/4,440; Mean (SD) age, 72.1 (5.6) years; Mean (SD) follow-up, 3.7 (0.6) years.

Model 1: adjusted for age, sex, ethnicity, marital status and occupational position.

Model 2: Model 1 + health behaviours.

Model 3: Model 2 + BMI categories and 9-point multimorbidity score.

Supplementary table D. Association between standardized measures of motor function and subsequent mortality as a function of multimorbidity status at assessment of motor function.

	**Participants without Multimorbidity*** HR (95% CI)	**Participants with Multimorbidity*** HR (95% CI)
**Motor function in 2007-2009† ‡**		
Walking speed	1.24 (1.11 to 1.37)	1.24 (1.04 to 1.47)
Grip strength	1.15 (1.04 to 1.27)	1.20 (1.02 to 1.42)
Timed chair rises	1.20 (1.10 to 1.30)	1.10 (0.95 to 1.27)
SF-36 PCS score	1.25 (1.14 to 1.37)	1.13 (0.99 to 1.29)
Limitations in ADL or IADL	1.23 (0.96 to 1.57)	1.62 (1.16 to 2.25)
**Motor function in 2012-2013† §**		
Walking speed	1.37 (1.17 to 1.60)	1.46 (1.20 to 1.78)
Grip strength	1.10 (0.96 to 1.27)	1.24 (1.04 to 1.48)
Timed chair rises	1.26 (1.12 to 1.43)	1.19 (1.06 to 1.34)
SF-36 PCS score	1.21 (1.07 to 1.36)	1.18 (1.02 to 1.37)
Limitations in ADL or IADL	1.37 (1.00 to 1.87)	1.55 (1.08 to 2.22)
**Motor function in 2015-2016† ¶**		
Walking speed	1.28 (0.97 to 1.69)	1.75 (1.37 to 2.25)
Grip strength	1.28 (1.00 to 1.64)	1.33 (1.04 to 1.70)
Timed chair rises	1.34 (1.14 to 1.58)	1.15 (1.02 to 1.29)
SF-36 PCS score	1.27 (1.04 to 1.55)	1.26 (1.05 to 1.51)
Limitations in ADL or IADL	1.37 (0.77 to 2.42)	1.97 (1.21 to 3.20)

Abbreviations: ADL: Activities of Daily Living; IADL: Instrumental Activities of Daily Living; SF-36 PCS score: Physical Component Summary score of the Short Form 36 General Health Survey; HR: Hazard ratio; CI: Confidence interval.

*Chronic conditions considered were: diabetes, coronary heart disease, stroke, cancer, dementia, Parkinson's disease, chronic obstructive pulmonary disease, depression, and arthritis; analyses adjusted for age, sex, ethnicity, marital status and occupational position, health behaviours, and BMI categories.

†HRs for mortality associated with 1 SD sex-specific poorer motor function, corresponding to 26.2 (25.4) cm/s slower walking speed, 8.5 (6.2) kg lower grip strength, 3.3 (3.6) more seconds to undertake timed chair rises and 8.0 (10.7) lower score in PCS in men (women) respectively. Limitations in ADL or IADL reflects having 1 or more limitations.

‡Multimorbidity free = N mortality/N total: 444/4,869; with multimorbidity = N mortality/N total: 166/776.

§Multimorbidity free = N mortality/N total: 228/4,204; with multimorbidity = N mortality/N total: 131/879.

¶Multimorbidity free = N mortality/N total: 74/3,569; with multimorbidity = N mortality/N total: 76/871.

Supplementary table E. Association between standardized measures of motor function and subsequent mortality, with additional adjustment for global cognition (Mini Mental State Examination).

	**Adjusted for all covariates***<br>HR (95% CI)
**Motor function in 2007-2009† ‡**	
Walking speed	1.20 (1.10 to 1.32)
Grip strength	1.14 (1.05 to 1.25)
Timed chair rises	1.15 (1.07 to 1.23)
SF-36 PCS score	1.16 (1.08 to 1.26)
Limitations in ADL or IADL	1.31 (1.07 to 1.59)
**Motor function in 2012-2013† §**	
Walking speed	1.38 (1.22 to 1.56)
Grip strength	1.14 (1.02 to 1.27)
Timed chair rises	1.19 (1.09 to 1.29)
SF-36 PCS score	1.17 (1.07 to 1.29)
Limitations in ADL or IADL	1.35 (1.07 to 1.72)
**Motor function in 2015-2016† ¶**	
Walking speed	1.36 (1.12 to 1.65)
Grip strength	1.22 (1.02 to 1.46)
Timed chair rises	1.13 (1.02 to 1.25)
SF-36 PCS score	1.19 (1.04 to 1.37)
Limitations in ADL or IADL	1.40 (0.96 to 2.03)

Abbreviations: ADL: Activities of Daily Living; IADL: Instrumental Activities of Daily Living; SF-36 PCS score: Physical Component Summary score of the Short Form 36 General Health Survey; HR: Hazard ratio; CI: Confidence interval.

*Models adjusted for age, sex, ethnicity, marital status and occupational position, health behaviours, BMI categories, 9-point multimorbidity score, and the Mini Mental State Examination score.

†HRs for mortality associated with 1 SD sex-specific poorer motor function, corresponding to 26.2 (25.4) cm/s slower walking speed, 8.5 (6.2) kg lower grip strength, 3.3 (3.6) more seconds to undertake timed chair rises and 8.0 (10.7) lower score in PCS in men (women) respectively. Limitations in ADL or IADL reflects having 1 or more limitations.

‡N mortality/N total = 591/5,501; Mean (SD) age, 65.6 (5.9) years; Mean (SD) follow-up, 10.6 (1.8) years.

§N mortality/N total = 349/4,956; Mean (SD) age, 69.4 (5.8) years; Mean (SD) follow-up, 6.8 (1.0) years.

¶N mortality/N total = 145/4,405; Mean (SD) age, 72.1 (5.6) years; Mean (SD) follow-up, 3.7 (0.6) years.

Supplementary table F. Association of decline in motor function performance between 2007-2009 and 2012-2013* and subsequent mortality.

	Model 1<br>HR (95% CI)	Model 2<br>HR (95% CI)	Model 3<br>HR (95% CI)
**Decline in motor function between 2007-2009 and 2012-2013* †**			
Walking speed	1.20 (1.06 to 1.34)	1.19 (1.06 to 1.34)	1.18 (1.05 to 1.32)
Grip strength	1.24 (1.06 to 1.45)	1.24 (1.06 to 1.45)	1.22 (1.04 to 1.42)
Chair rise time	1.11 (1.00 to 1.24)	1.10 (0.99 to 1.23)	1.08 (0.97 to 1.20)
SF-36 PCS score	1.19 (1.06 to 1.33)	1.16 (1.04 to 1.30)	1.16 (1.03 to 1.29)
**Change in limitations in ADL or IADL between 2007-2009 and 2012-2013**			
No limitations at both waves‡	Ref.	Ref.	Ref.
No limitations to 1 or more limitations§	1.62 (1.19 to 2.21)	1.52 (1.11 to 2.08)	1.37 (1.00 to 1.87)
1 or more limitations to no limitations¶	1.18 (0.73 to 1.92)	1.13 (0.70 to 1.84)	0.99 (0.61 to 1.61)
1 or more limitations at both waves II	1.74 (1.26 to 2.40)	1.61 (1.16 to 2.24)	1.32 (0.95 to 1.85)

Abbreviations: ADL: Activities of Daily Living; IADL: Instrumental Activities of Daily Living; SF-36 PCS score: Physical Component Summary score of the Short Form 36 General Health Survey; HR: Hazard ratio; CI: Confidence interval.

*Measures of motor function from 2007-2009 and 2013-2013 measures were standardized using mean and SD from 2007-2009, separately in men and women, so that estimates represent HRs associated with a 1SD decline in motor function performance between 2007-2009 and 2012-2013 for all tests except "limitations in ADL or IADL" which was dichotomized using 1 or more limitations. This corresponds to a decline of 26.2 (25.4) cm/s in walking speed, 8.5 (6.2) kg in grip strength, 8.0 (10.7) in PCS score, and slowing by 3.3 (3.6) seconds in timed chair rise, among men (women) respectively.

†N mortality/N total = 316/4,606; Mean (SD) age, 68.4 (5.7) years; Mean (SD) follow-up, 7.0 (1.0) years.

‡N mortality/N total = 202/3,567.

§N mortality/N total = 50/406.

¶N mortality/N total = 46/353.

II N mortality/N total = 18/280.

Model 1: adjusted for age, sex, ethnicity, marital status and occupational position.
Model 2: Model 1 + health behaviours.
Model 3: Model 2 + BMI categories and 9-point multimorbidity score.

Supplementary table G. Population characteristics in 2007-2009 by survival status at time 0 in the retrospective time scale in the analysis (date of death or 31st of December 2017).

		Vital status, 31st December 2017	
	**Total** (N= 5,645)	**Decedents** (N = 458)	**Survivors** (N = 5,187)
Age, M (SD)	65.6 (5.9)	69.7 (6.0)	65.3 (5.7)
Women	1,539 (27.3)	111 (24.2)	1,428 (27.5)
White ethnicity	5,244 (92.9)	430 (93.9)	4,814 (92.8)
Living in couple	4,263 (75.5)	311 (67.9)	3,952 (76.2)
High socioeconomic position	2,476 (43.9)	177 (38.6)	2,299 (44.3)
Moderate alcohol consumption	2,901 (51.4)	212 (46.3)	2,689 (51.8)
Never smoker	2,722 (48.2)	198 (43.2)	2,524 (48.7)
Daily fruit & vegetable consumption	2,267 (40.2)	183 (40.0)	2,084 (40.2)
Physical activity at recommended levels	3,236 (57.3)	226 (49.3)	3.010 (58.0)
**Motor function**			
Walking speed (cm/s), M (SD)	110.6 (26.7)	101.2 (28.2)	111.5 (26.3)
Grip strength (kg), M (SD)	38.0 (10.6)	35.1 (10.5)	38.3 (10.6)
Chair rise time (s), M (SD)	11.3 (3.4)	12.5 (4.3)	11.2 (3.3)
SF-36 PCS score	48.8 (8.7)	45.2 (10.1)	49.1 (8.5)
Limitations in ADL or IADL	860 (15.2)	109 (23.8)	751 (14.5)
**BMI, M (SD)**	26.7 (4.4)	26.7 (4.7)	26.7 (4.4)
**Chronic conditions**			
Diabetes	541 (9.6)	58 (12.7)	483 (9.3)
Coronary Heart Disease	1,167 (20.7)	136 (29.7)	1,031 (19.9)
Stroke	216 (3.8)	45 (9.8)	171 (3.3)
Cancer	436 (7.7)	89 (19.4)	347 (6.7)
Dementia	7 (0.1)	2 (0.4)	5 (0.1)
Parkinson's disease	20 (0.4)	6 (1.3)	14 (0.3)
Chronic Obstructive Pulmonary Disease	47 (0.8)	14 (3.1)	33 (0.6)
Depression	561 (9.9)	54 (11.8)	507 (9.8)
Arthritis	496 (8.8)	48 (10.5)	448 (8.6)
**Multimorbidity score***			
0	3,098 (54.9)	153 (33.4)	2,945 (56.8)
1	1,771 (31.4)	181 (39.5)	1,590 (30.7)
2 or more	776 (13.8)	124 (27.1)	652 (12.3)

Abbreviations: M, mean; SD, standard deviation; SF-36: SF-36 PCS score: Physical Component Summary score of the Short Form 36 General Health Survey; ADL: Activities of Daily Living; IADL: Instrumental Activities of Daily Living.
Data are N (%) unless stated otherwise.
*****The score is composed of the nine chronic conditions listed above at the exception of obesity.

Supplementary table H. Differences in motor function between survivors and decedents in the 10 years preceding death in analysis adjusted only for sociodemographic variables, N mortality/N total = 484/6,194.* †

	OBJECTIVE MEASURES						SELF-REPORTED MEASURES			
	Walking speed		Grip strength		Chair rise time		SF-36 PCS score		ADL/IADL limitations	
**Years preceding death**	**Difference in mean (95% CI)**	***p***	**Difference in mean (95% CI)**	***p***	**Difference in mean (95% CI)**	***p***	**Difference in mean (95% CI)**	***p***	**Difference in probabilities (95% CI)**	***P***
**-10**	0.21 (-0.01 to 0.44)	0.063	0.03 (-0.17 to 0.24)	0.76	0.41 (0.17 to 0.64)	0.001	0.12 (-0.11 to 0.34)	0.307	0.04 (0.05 to 0.12)	0.39
**-9**	0.26 (0.10 to 0.42)	0.001	0.07 (-0.08 to 0.22)	0.36	0.36 (0.19 to 0.52)	< 0.001	0.16 (0.00 to 0.31)	0.054	0.03 (0.02 to 0.09)	0.25
**-8**	0.30 (0.19 to 0.42)	< 0.001	0.10 (-0.01 to 0.21)	0.09	0.33 (0.21 to 0.45)	< 0.001	0.20 (0.08 to 0.32)	0.001	0.04 (0.01 to 0.08)	0.11
**-7**	0.34 (0.24 to 0.44)	< 0.001	0.12 (0.02 to 0.22)	0.02	0.32 (0.22 to 0.42)	< 0.001	0.24 (0.14 to 0.35)	< 0.001	0.04 (0.00 to 0.08)	0.04
**-6**	0.37 (0.28 to 0.47)	< 0.001	0.14 (0.04 to 0.23)	0.007	0.34 (0.23 to 0.44)	< 0.001	0.29 (0.19 to 0.39)	< 0.001	0.05 (0.01 to 0.09)	0.02
**-5**	0.40 (0.30 to 0.49)	< 0.001	0.14 (0.04 to 0.24)	0.005	0.37 (0.27 to 0.48)	< 0.001	0.34 (0.24 to 0.45)	< 0.001	0.06 (0.02 to 0.10)	0.006
**-4**	0.41 (0.32 to 0.50)	< 0.001	0.14 (0.04 to 0.23)	0.004	0.43 (0.33 to 0.53)	< 0.001	0.40 (0.29 to 0.50)	< 0.001	0.07 (0.03 to 0.11)	0.001
**-3**	0.43 (0.34 to 0.51)	< 0.001	0.13 (0.04 to 0.22)	0.006	0.51 (0.41 to 0.61)	< 0.001	0.45 (0.35 to 0.55)	< 0.001	0.09 (0.05 to 0.13)	< 0.001
**-2**	0.43 (0.34 to 0.52)	< 0.001	0.11 (0.01 to 0.20)	0.03	0.61 (0.50 to 0.73)	< 0.001	0.51 (0.41 to 0.62)	< 0.001	0.12 (0.07 to 0.17)	< 0.001
**-1**	0.43 (0.31 to 0.55)	< 0.001	0.08 (-0.04 to 0.20)	0.19	0.74 (0.59 to 0.89)	< 0.001	0.58 (0.44 to 0.72)	< 0.001	0.15 (0.08 to 0.22)	< 0.001
**0**	0.42 (0.24 to 0.60)	< 0.001	0.05 (-0.13 to 0.22)	0.61	0.88 (0.67 to 1.10)	< 0.001	0.64 (0.44 to 0.84)	< 0.001	0.19 (0.08 to 0.30)	0.001
**Difference in trajectories**	**0.15**		**0.56**		**< 0.001**		**< 0.001**		**0.04**	

Abbreviations: ADL: Activities of Daily Living; IADL: Instrumental Activities of Daily Living; SF-36 PCS score: Physical Component Summary score of the Short Form 36 General Health Survey; CI: Confidence interval.

* Greater the estimated mean difference poorer the motor function among decedents compared to survivors, apart for ADL/IADL limitations that reflect probability of one or more limitations. 1 SD sex-specific motor function corresponded to 26.2 (25.4) cm/s slower walking speed, 8.5 (6.2) kg lower grip strength, 3.3 (3.6) more seconds to undertake timed chair rises and 8.0 (10.7) lower score in PCS in men (women) respectively.

†Estimated from linear mixed models except ADL/IADL limitations where logistic regression with generalized estimated equation models were used; analyses adjusted for age at year 0, sex, ethnicity, marital status, occupational position, vital status, time terms (time & time^2), interactions of sociodemographic covariates with time terms.

Supplementary table I. Difference in the probability of limitations in ADL and IADL, examined separately, between survivors and decedents in the 10 years preceding death, N mortality/N total = 484/6,194.*

	**ADL limitations**		**IADL limitations**	
**Years preceding death**	**Difference in probability (%) (95% CI)**	**p**	**Difference in probability (%) (95% CI)**	**p**
-10	0.00 (-0.02 to 0.03)	0.69	-0.01 (-0.03 to 0.02)	0.57
-9	0.00 (-0.01 to 0.02)	0.55	-0.00 (-0.02 to 0.02)	0.82
-8	0.01 (-0.01 to 0.02)	0.36	-0.00 (-0.01 to 0.02)	0.73
-7	0.01 (-0.00 to 0.02)	0.20	0.01 (-0.01 to 0.02)	0.28
-6	0.01 (-0.00 to 0.02)	0.11	0.01 (-0.00 to 0.03)	0.11
-5	0.01 (-0.00 to 0.02)	0.06	0.02 (-0.00 to 0.04)	0.06
-4	0.02 (0.00 to 0.03)	0.02	0.02 (-0.00 to 0.04)	0.03
-3	0.02 (0.01 to 0.04)	0.002	0.03 (0.01 to 0.04)	0.01
-2	0.03 (0.01 to 0.05)	< 0.001	0.03 (0.01 to 0.05)	0.01
-1	0.04 (0.01 to 0.07)	0.003	0.03 (-0.00 to 0.06)	0.08
0	0.05 (0.01 to 0.10)	0.03	0.02 (-0.02 to 0.07)	0.31
**Difference in trajectories**	**0.07**		**0.30**	

Abbreviations: ADL: Activities of Daily Living; IADL: Instrumental Activities of Daily Living; CI: Confidence interval.

***** Estimates from logistic regression with generalized estimated equation; analyses adjusted for age, sex, ethnicity, marital status, occupational position, vital status, time terms (time, time^2), interactions of these covariates with time terms, and health behaviours, BMI categories and 9-point multimorbidity score assessed at motor function measurement. Higher differences reflect higher probabilities of ADL/IADL limitations among decedents than survivors.

Supplementary table J. Difference in motor function between survivors and decedents in the 10 years preceding death with additional adjustment for global cognition (Mini Mental State Examination), N mortality/N total = 477/6,149.* †

	OBJECTIVE MEASURES						SELF-REPORTED MEASURES			
	Walking speed		Grip strength		Chair rise time		SF-36 PCS score		ADL/IADL limitations	
**Years preceding death**	**Difference in mean (95% CI)**	***p***	**Difference in mean (95% CI)**	***p***	**Difference in mean (95% CI)**	***p***	**Difference in mean (95% CI)**	***p***	**Difference in probabilities (95% CI)**	***P***
**-10**	0.15 (-0.07 to 0.37)	0.19	0.00 (-0.21 to 0.20)	0.99	0.36 (0.1 to 0.602)	0.003	0.00 (-0.22 to 0.22)	0.99	0.01 (-0.03 to 0.05)	0.69
**-9**	0.21 (0.05 to 0.36)	0.01	0.04 (-0.11 to 0.19)	0.61	0.31 (0.15 to 0.48)	< 0.001	0.05 (-0.10 to 0.21)	0.49	0.01 (-0.02 to 0.03)	0.61
**-8**	0.25 (0.14 to 0.37)	< 0.001	0.07 (-0.04 to 0.18)	0.23	0.28 (0.16 to 0.40)	< 0.001	0.11 (-0.01 to 0.22)	0.07	0.01 (-0.01 to 0.03)	0.48
**-7**	0.29 (0.19 to 0.39)	< 0.001	0.09 (-0.01 to 0.19)	0.07	0.27 (0.17 to 0.38)	< 0.001	0.16 (0.06 to 0.26)	0.002	0.01 (-0.01 to 0.02)	0.34
**-6**	0.32 (0.23 to 0.41)	< 0.001	0.10 (0.01 to 0.20)	0.04	0.28 (0.18 to 0.39)	< 0.001	0.21 (0.11 to 0.31)	< 0.001	0.01 (-0.01 to 0.03)	0.22
**-5**	0.34 (0.25 to 0.43)	< 0.001	0.11 (0.01 to 0.21)	0.03	0.31 (0.21 to 0.42)	< 0.001	0.25 (0.15 to 0.35)	< 0.001	0.01 (-0.00 to 0.03)	0.12
**-4**	0.35 (0.26 to 0.44)	< 0.001	0.10 (0.01 to 0.20)	0.03	0.37 (0.26 to 0.47)	< 0.001	0.30 (0.20 to 0.40)	< 0.001	0.02 (0.00 to 0.04)	0.04
**-3**	0.35 (0.27 to 0.43)	< 0.001	0.09 (0.00 to 0.18)	0.05	0.44 (0.34 to 0.54)	< 0.001	0.35 (0.25 to 0.44)	< 0.001	0.03 (0.01 to 0.05)	0.004
**-2**	0.34 (0.25 to 0.43)	< 0.001	0.07 (-0.03 to 0.16)	0.17	0.53 (0.42 to 0.64)	< 0.001	0.39 (0.29 to 0.50)	< 0.001	0.04 (0.02 to 0.06)	0.001
**-1**	0.32 (0.20 to 0.45)	< 0.001	0.04 (-0.09 to 0.16)	0.57	0.64 (0.49 to 0.78)	< 0.001	0.44 (0.30 to 0.57)	< 0.001	0.05 (0.02 to 0.09)	0.004
**0**	0.30 (0.12 to 0.48)	0.001	-0.01 (-0.18 to 0.17)	0.95	0.77 (0.56 to 0.98)	< 0.001	0.48 (0.28 to 0.68)	< 0.001	0.08 (0.01 to 0.14)	0.03
**Difference in trajectories**	**0.28**		**0.47**		**< 0.001**		**< 0.001**		**0.09**	

Abbreviations: ADL: Activities of Daily Living; IADL: Instrumental Activities of Daily Living; SF-36 PCS score: Physical Component Summary score of the Short Form 36 General Health Survey; CI: Confidence interval.

* Greater the estimated mean difference poorer the motor function among decedents compared to survivors, apart for ADL/IADL limitations that reflect probability of one or more limitations. 1 SD sex-specific motor function corresponded to 26.2 (25.4) cm/s slower walking speed, 8.5 (6.2) kg lower grip strength, 3.3 (3.6) more seconds to undertake timed chair rises and 8.0 (10.7) lower score in PCS in men (women) respectively.

†Estimated from linear mixed models except ADL/IADL limitations where logistic regression with generalized estimated equation models were used; analyses adjusted for age at year 0, sex, ethnicity, marital status, occupational position, vital status, time terms (time & time^2), interactions of sociodemographic covariates with time terms, and health behaviours, BMI categories, 9-point multimorbidity score and the Mini Mental State Examination score assessed at motor function measurement.
